# Comment on "The clinical significance of D-dimer concentrations predicting the risk of venous thromboembolism in patients with hyperemesis gravidarum"

**DOI:** 10.1590/1806-9282.20251376

**Published:** 2025-12-08

**Authors:** Emin Barbarus

**Affiliations:** 1 Balıkesir University, Department of Cardiovascular Surgery – Balıkesir, Turkey.

Dear Editor,

I read with interest the study conducted by Kadıoglu et al. and published in your journal under the title "The clinical significance of D-dimer concentrations predicting the risk of venous thromboembolism in patients with hyperemesis gravidarum."^1^ The study compared serum D-dimer and fibrinogen levels in pregnant women diagnosed with hyperemesis gravidarum (HG) to those in healthy pregnant women. While the topic is clinically significant, I would like to share my concerns regarding the study design, data interpretation, and the strength of the conclusions reached.

D-dimer is a fibrin degradation product, and its levels may increase in many physiological and pathological conditions. In the diagnosis of venous thromboembolism (VTE), although its positive predictive value is limited, it is widely used to rule out the diagnosis due to its high negative predictive value^2^. In this regard, according to guidelines, the predictive value of D-dimer levels is limited^3^. Additionally, in clinical practice, D-dimer is primarily used to monitor treatment response, determine the duration of anticoagulant therapy, and assess the need for extended treatment. In the present study, the predictive value of D-dimer levels for VTE in cases of HG was investigated. However, since no cases of VTE developed in either group, it was not possible to establish a relationship between D-dimer levels and the presence of VTE, which should be considered an important methodological limitation of the study.

In conclusion, although the study addresses an original and interesting topic, methodological limitations require a cautious approach to the results. I hope that my comments will contribute constructively to your journal's scientific contribution process.

Sincerely,

## Data Availability

The datasets generated and/or analyzed during the current study are available from the corresponding author upon reasonable request.
